# Intrapartum exposure to synthetic oxytocin, maternal BMI, and neurodevelopmental outcomes in children within the ECHO consortium

**DOI:** 10.1186/s11689-024-09540-1

**Published:** 2024-05-26

**Authors:** Lisa Kurth, T. Michael O’Shea, Irina Burd, Anne L. Dunlop, Lisa Croen, Greta Wilkening, Ting-ju Hsu, Stephan Ehrhardt, Arvind Palanisamy, Monica McGrath, Marie L. Churchill, Daniel Weinberger, Marco Grados, Dana Dabelea

**Affiliations:** 1 Department of Pediatrics, Developmental Section, University of Colorado School of Medicine, 13123 E. 16th Ave. B065, Aurora, CO 80045 USA; 2 Department of Pediatrics, University of North Carolina School of Medicine, Chapel Hill, NC USA; 3 Departments of Obstetrics, Gynecology and Reproductive Sciences, University of Maryland School of Medicine, Baltimore, MD USA; 4 Department of Gynecology and Obstetrics, Emory University School of Medicine, Atlanta, GA USA; 5 Kaiser Permanente Division of Research, Northern California, Oakland, CA USA; 6 Department of Pediatrics, University of Colorado School of Medicine, Aurora, CO USA; 7 Department of Epidemiology, Johns Hopkins Bloomberg School of Public Health, Baltimore, MD USA; 8 Department of Anesthesiology, Washington University School of Medicine in St. Louis, St. Louis, MO USA; 9 Departments of Psychiatry, Neurology, Neuroscience, Johns Hopkins School of Medicine, Baltimore, MD USA; 10 The Lieber institute for Brain Development, Baltimore, MD USA; 11 Departments of Psychiatry and Behavioral Sciences, Johns Hopkins School of Medicine, Baltimore, MD USA; 12 Kennedy Krieger Institute, Baltimore, MD USA; 13 Lifecourse Epidemiology of Adiposity and Diabetes (LEAD) Center, University of Colorado Anschutz Medical Campus, Aurora, CO USA

**Keywords:** Neurodevelopment, ADHD, Autism, ASD, Synthetic oxytocin, Obesity, BMI

## Abstract

**Background:**

Synthetic oxytocin (sOT) is frequently administered during parturition. Studies have raised concerns that fetal exposure to sOT may be associated with altered brain development and risk of neurodevelopmental disorders. In a large and diverse sample of children with data about intrapartum sOT exposure and subsequent diagnoses of two prevalent neurodevelopmental disorders, i.e., attention deficit hyperactivity disorder (ADHD) and autism spectrum disorder (ASD), we tested the following hypotheses: (1) Intrapartum sOT exposure is associated with increased odds of child ADHD or ASD; (2) associations differ across sex; (3) associations between intrapartum sOT exposure and ADHD or ASD are accentuated in offspring of mothers with pre-pregnancy obesity.

**Methods:**

The study sample comprised 12,503 participants from 44 cohort sites included in the Environmental Influences on Child Health Outcomes (ECHO) consortium. Mixed-effects logistic regression analyses were used to estimate the association between intrapartum sOT exposure and offspring ADHD or ASD (in separate models). Maternal obesity (pre-pregnancy BMI ≥ 30 kg/m^2^) and child sex were evaluated for effect modification.

**Results:**

Intrapartum sOT exposure was present in 48% of participants. sOT exposure was not associated with increased odds of ASD (adjusted odds ratio [aOR] 0.86; 95% confidence interval [CI], 0.71–1.03) or ADHD (aOR 0.89; 95% CI, 0.76–1.04). Associations did not differ by child sex. Among mothers with pre-pregnancy obesity, sOT exposure was associated with lower odds of offspring ADHD (aOR 0.72; 95% CI, 0.55–0.96). No association was found among mothers without obesity (aOR 0.97; 95% CI, 0.80–1.18).

**Conclusions:**

In a large, diverse sample, we found no evidence of an association between intrapartum exposure to sOT and odds of ADHD or ASD in either male or female offspring. Contrary to our hypothesis, among mothers with pre-pregnancy obesity, sOT exposure was associated with lower odds of child ADHD diagnosis.

**Supplementary Information:**

The online version contains supplementary material available at 10.1186/s11689-024-09540-1.

## Background

For over 50 years, synthetic oxytocin (sOT), an exogenous neuropeptide and uterine stimulant (trade names Pitocin® and Syntocinon®), typically administered to the pregnant individual by intravenous infusion, has been increasingly used as a first line approach to induce and/or augment labor by stimulating uterine contractions [1–6]. Administration of sOT as a single agent for labor induction and/or augmentation assists in the expulsion of the fetus in the setting of childbirth complications [7] and may minimize risk of instrumental deliveries [8]. However, despite the increasing frequency with which sOT is administered to pregnant women [9–11], only a few large studies have characterized the relationship of intrapartum sOT and child neurodevelopmental outcome. One of the largest studies (*n* = 1.5 million), based on a national cohort of Scandinavian children, found an approximately 20% increased risk of attention deficit hyperactivity disorder (ADHD) and autism spectrum disorder (ASD) associated with sOT exposure. However, authors were reassured regarding clinical use of sOT as confounder adjustment attenuated this association [12].

Child neurodevelopmental outcomes following intrapartum sOT exposure have not been studied in large samples of children born in the United States (US) [13, 14], where obstetric medical practices may differ from those of other countries [15]. Among existing studies, some report associations between sOT exposure and ADHD and/or ASD [13, 14, 16–19], some report mixed results [20–25], and some report no associations [12, 26–29]. Preclinical models provide evidence of potential neuroprotective effects of endogenous oxytocin; however, if pulsatile uterine contractions are excessively prolonged by treatment with exogenous sOT, uteroplacental perfusion can be reduced to an extent sufficient to alter brain development [30]. Thus, a greater understanding is needed regarding the relationship of fetal intrapartum exposure to sOT and the risk(s) of child neurodevelopmental outcomes.

ADHD and ASD are among the most prevalent neurodevelopmental disorders with poorly understood etiology. ADHD, a disorder characterized by symptoms of inattention, distractibility, impulsivity, hyperactivity and behavioral dysregulation [31], affects almost 10% of US children [32, 33]. ASD, characterized by deficits in social interaction and social communications with restricted or repetitive patterns of behavior and interests [34], affects 1 in 36 [35] eight-year-old US children [36]. ADHD and ASD demonstrate high diagnostic comorbidity [37], and represent the two most prevalent developmental disabilities among children aged 3 to 17 years in the US and other high-income countries [38, 39]. In addition, the unique constellation of behavioral characteristics typified by children diagnosed with ADHD and/or ASD have long posed significant burdens within the familial and educational settings [40–43]. Importantly, the steadily rising prevalence of both ADHD and ASD impel an urgent need to identify modifiable risk factors [44–48]. The poorly understood etiology, comorbidity, and prevalence of ADHD and ASD prompted our examination of the association between intrapartum sOT exposure and these specific neurodevelopmental conditions.

Because females and males differ with respect to neurodevelopmental vulnerability [17, 49] and males experience increased risk of both ADHD and ASD [50], we evaluated sex differences in the associations between sOT and neurodevelopmental outcomes. In addition, because mothers with obesity exhibit poor uterine contractility as compared to non-obese mothers, and therefore often require sOT induction to facilitate labor (50–53), we evaluated maternal pre-pregnancy obesity (e.g. BMI) as a potential effect measure modifier [51]. Here we tested three hypotheses: (1) Intrapartum exposure to sOT is associated with increased odds of child ADHD or ASD; (2) associations differ across sex; (3) associations between intrapartum sOT exposure and ADHD or ASD would be accentuated in offspring of mothers with pre-pregnancy obesity.

## Methods

### Data source

We used data from a large consortium, the Environmental influences on Child Health Outcomes (ECHO) program, to evaluate the association between intrapartum sOT and offspring ADHD and ASD. The ECHO program is a consortium of longitudinal cohort studies established by the National Institutes of Health (NIH) to examine the impacts of various exposures – chemical, biological, physical, and social – in relation to child health and development [52]. Specifically, ECHO research focuses on childbirth and perinatal outcomes, respiratory illness, obesity, neurodevelopment, and overall wellness, relying on a protocol of harmonized derived variables among cohort sites [53–55]. The study protocol was approved by the cohort-specific and/or the single ECHO Institutional Review Boards. Written informed consent was obtained for ECHO Cohort Data Collection Protocol participation and for participation in specific cohorts.

The study population included 12,503 biological mother/child pairs enrolled in 44 ECHO cohorts. The 44 cohorts included two ASD-enriched studies, six cohorts enrolling children from neonatal intensive care units (NICU), and thirty-six general population cohorts (See Additional File 1 Table S1 and Table S2). ASD-enriched studies included children originally enrolled as part of a case-control study of ASD, developmental delays, and typical development as well as a cohort enrolling younger siblings of children with ASD. NICU cohorts enrolled directly from NICUs. General population cohorts consisted of pregnancy and early-childhood studies evaluating other child health outcomes, including birth outcomes, growth and development, asthma, and overall wellbeing. Inclusion criteria for the study were (1) singleton births; (2) data available on child ADHD and ASD diagnoses, and (3) data on maternal administration of sOT during labor or delivery. For families with more than one child enrolled in the ECHO cohort, one sibling was randomly selected to be included in this study. We restricted inclusion to those cohorts with available data on at least 20 mother/child dyads. The decision-logic for inclusion and exclusion of cohorts and participants is displayed in Additional File 1 Fig. S1. We identified 1073 ADHD cases and 851 ASD cases in our study population.

### Synthetic oxytocin administration

Synthetic oxytocin use during childbirth (yes vs. no) was ascertained from either medical record abstraction or self-report by the mother. Regarding forms of terminology used to search the ECHO platform to identify relevant data included for harmonization of extant and new data (related to intrapartum sOT use), the following terms were included: sOT, Oxytocin, Pitocin, Syntocinon, uterotonic, uterine stimulant, stimulation, induction, induce, augmentation, augment. Terminology on the ECHO forms were oxytocin and Pitocin. Use of sOT for each mother-child pair was ascertained based on a prioritization of available information for use in the following order: (1) documentation of sOT administration during labor and delivery in maternal medical records, (2) documentation of labor induction or augmentation in maternal medical records, (3) documentation of labor induction or augmentation in childbirth medical records, and (4) maternal self-report of having been administered sOT.

### ADHD and ASD

We defined ADHD and ASD based on caregiver report of physician-diagnosed disorders. Caregivers were asked whether a doctor or other health care provider had ever informed them that their child has or had Attention Deficit Disorder (ADD) or Attention Deficit /Hyperactivity Disorder (ADHD) for an ADHD diagnosis, and/or ASD Spectrum Disorder (ASD), Asperger’s Disorder or Pervasive Developmental Disorder (PDD) for an ASD diagnosis. In some cohorts, ASD diagnosis was obtained by utilizing several clinical sources, including established gold-standard diagnostic instruments, such as the *Autism Diagnostic Observation Schedule* [56] or a diagnosis extracted from medical records.

### Covariates

Self-reported maternal races were defined as American Indian/Alaskan Native, Asian, Black, Native Hawaiian or Pacific Islander, White, Other Race, and Multiple Races. Mother’s highest education was categorized as high school degree or equivalent or less; some college with no degree; and bachelor’s degree and above. Child characteristics include caregiver-reported child race, childbirth year (< 2005; 2006–2010; 2011–2015; 2016–2022), and child sex assigned at birth (male or female).

Maternal age at the time of delivery was determined from demographic questionnaires and maternal medical records. Preterm birth (yes/no), defined as birth prior to 37 weeks gestation, was based on available reports for gestational age.

Gestational age at birth in completed weeks was obtained through abstraction of maternal or child medical records or through parent-report. For medical record abstraction, an accepted hierarchy [57, 58] was employed to ascertain the most accurate measure for estimating the due date: dating based on embryo placement following in vitro fertilization or dating based on artificial insemination, obstetrical estimate from first trimester ultrasound; obstetrical estimated from ultrasound taken in the second trimester with fetal biparietal diameter dating within 2 weeks of sure last menstrual period (LMP); ultrasound taken in the second trimester with unsure or no LMP date; report from obstetrical medical record reporting “consensus” estimated date of delivery with no ultrasound documented during first and second trimester; obstetrical estimate from LMP only; neonatal estimate of gestational age at birth obtained from child medical records; estimated from cohort research encounter; reported by mother; and estimated on cohort-provided estimated date of delivery without further description.

Large for gestational age (LGA), defined as child birthweight-for-gestational age and sex > 90th percentile (percentiles derived from the International Fetal and Newborn Growth Consortium for the 21st Century [INTERGROWTH-21]) [59] was calculated. Pre-pregnancy obesity was defined as a body mass index (BMI) *≥* 30 kg/m^2^ according to accepted definitions [35]. Pre-pregnancy BMI was obtained using measured or self-reported height and weight between 12 months prior to conception through the first trimester. Gestational diabetes mellitus (GDM) was defined as new-onset diabetes during pregnancy based on self-report or as indicated in maternal medical records.

### Statistical analysis

We compared the distribution of demographic characteristics and medical conditions between women who received sOT during labor and delivery and those who did not using Pearson chi-square tests. Using mixed-effects logistic models (“glmer” function from the “lme4” R package), we calculated unadjusted and covariate-adjusted odds ratios (aORs) and corresponding 95% confidence intervals (CI) to estimate associations between sOT use during childbirth and risk of ADHD or ASD in the offspring. Models were fitted with maximum likelihood estimators. Wald 95% CIs were constructed, and *P*-values were derived from the Wald z-test. In multivariable analyses, we adjusted for child race, ethnicity, sex, child’s birth year, gestational age and LGA status at birth, maternal age at delivery, and highest maternal education level. Maternal obesity prior to pregnancy and GDM were added to the adjusted model as covariates independently and in tandem. Models were fitted with random effects for individual cohorts to account for clustering within cohort. Based on a priori hypotheses that there would be variation by child sex and maternal pre-pregnancy obesity, fully adjusted models for both ADHD and ASD were stratified to examine for differences by strata. We evaluated effect modification by sex and by maternal pre-pregnancy obesity using product terms, sOT x sex, and sOT x maternal pre-pregnancy obesity. For all analyses, the criterion for statistical significance was *P* < 0.05, without adjustment for multiple comparisons.

Imputation was performed for missing data using multiple imputation by chained equations from the “mice” R package [60]. The results were pooled after 25 imputations with a maximum of 10 iterations. The imputation models included our variables of interest with cohort type (general population, NICU, or ASD-enriched) and individual cohort membership as classification variables. Regression estimates from the imputed datasets were pooled together using Rubin’s rule.

In a set of sensitivity analyses, we explored potential cohort effects by assessing whether observed associations between the sOT use and odds of ADHD or ASD differed after removing individual cohorts and/or cohort types based on specific enrollment criteria (e.g. ASD-enriched, NICU, and general population cohorts). All analyses were performed using the R statistical software package, version 4.1.0 (R Foundation for Statistical Computing, Vienna, Austria).

## Results

### Associations between participant characteristics and sOT exposure

Forty-eight percent of study participants were exposed to sOT. Table 1 shows socio-demographic characteristics of the sample by sOT exposure status. Maternal age at delivery and child sex assigned at birth were similar in sOT exposed mothers compared with those not exposed. Mean child age at diagnosis for ADHD was 7.10 in the sOT exposed group vs. 6.81 in the non-exposed group. Mean child age at diagnosis for ASD was 3.0 in the sOT exposed group, vs. 3.86 in the non-exposed group. Children exposed to sOT were more likely to be Hispanic (24.5% vs. 20.5%), and less likely to be White (56.7% vs. 60.9%) and born preterm (9.1% vs. 20.2%). Exposed mothers were more likely to have pre-pregnancy obesity (28.8% vs. 26.7%) and GDM (9.0% vs. 7.2%) compared with those not exposed.

**Table 1:** Characteristics of children and mothers according to sOT exposure status, ECHO study (*N* = 12,503)

	sOT exposed *n* (%)	sOT unexposed *n* (%)	*P*-value
Total	6,014 (48%)	6,489 (52%)	
*Child characteristics*			
ASD diagnosis, N (%) with data			0.001
No	5,558 (92.42%)	6,094 (93.91%)	
Yes	456 (7.58%)	395 (6.09%)	
Missing	0	0	
Child age at ASD diagnosis			< 0.001
Mean (SD)	3.00 (2.49)	3.86 (3.00)	
Missing	35	44	
ADHD diagnosis, N (%) with data			< 0.001
No	5,586 (92.88%)	5,844 (90.06%)	
Yes	428 (7.12%)	645 (9.94%)	
Missing	0	0	
Child age at ADHD diagnosis			0.120
Mean (SD)	7.10 (2.93)	6.81 (2.66)	
Missing	54	54	
Child sex at birth, N (%) with data			0.221
Male	3,190 (53.04%)	3,370 (51.93%)	
Female	2,824 (46.96%)	3,119 (48.07%)	
Missing	0	0	
Child race, N (%) with data			< 0.001
American Indian or Alaska Native	56 (< 1%)	148 (2.35%)	
Asian	345 (5.94%)	249 (3.96%)	
Black	1,135 (19.55%)	1,197 (19.02%)	
Multiple race	765 (13.18%)	676 (10.74%)	
Native Hawaiian or other Pacific Islander	21 (< 1%)	11 (< 1%)	
Other race	192 (3.31%)	180 (2.86%)	
White	3,292 (56.7%)	3,833 (60.9%)	
Missing	208	195	
Child ethnicity, N (%) with data			< 0.001
Hispanic	1,464 (24.53%)	1,321 (20.47%)	
Non-Hispanic	4,504 (75.47%)	5,133 (79.53%)	
Missing	46	35	
Preterm birth, N (%) with data			< 0.001
Yes	546 (9.09%)	1,311 (20.23%)	
No	5,461 (90.91%)	5,168 (79.77%)	
Missing	7	10	
Gestational age at birth (weeks)			< 0.001
Mean (SD)	38.60 (2.56)	37.16 (4.50)	
Missing	23	31	
Large for gestational age, N (%) with data			0.658
Yes	969 (16.29%)	1,052 (16.6%)	
No	4,980 (83.71%)	5,285 (83.4%)	
Missing	65	152	
Child year of birth, N (%) with data			< 0.001
<2005	441 (7.33%)	495 (7.63%)	
2006–2010	1,075 (17.87%)	1,231 (18.97%)	
2011–2015	1,775 (29.51%)	2,630 (40.53%)	
2016–2022	2,723 (45.28%)	2,133 (32.87%)	
Missing	0	0	
*Maternal characteristics*			
Maternal age at delivery			0.524
Mean (SD)	29.84 (5.61)	29.91 (5.62)	
Missing	18	31	
Maternal race, N (%) with data			< 0.001
American Indian or Alaska Native	55 (< 1%)	170 (2.75%)	
Asian	473 (8.35%)	328 (5.31%)	
Black	1,125 (19.85%)	1,182 (19.14%)	
Multiple race	306 (5.4%)	297 (4.81%)	
Native Hawaiian or other Pacific Islander	27 (< 1%)	17 (< 1%)	
Other race	182 (3.21%)	170 (2.75%)	
White	3,499 (61.74%)	4,013 (64.97%)	
Missing	347	312	
Maternal ethnicity, N (%) with data			< 0.001
Hispanic	1,199 (20.12%)	1,090 (16.96%)	
Non-Hispanic	4,759 (79.88%)	5,337 (83.04%)	
Missing	56	62	
Highest maternal education ever assessed, N (%) with data			0.014
High school degree, GED or equivalent and below	959 (16.13%)	1,149 (18.1%)	
Some college, no degree; Associate’s degree	1,771 (29.79%)	1,822 (28.7%)	
Bachelor’s degree and above	3,214 (54.07%)	3,378 (53.21%)	
Missing	70	140	
Pre-pregnancy obesity, N (%) with data			0.014
Yes	1,553 (28.75%)	1,548 (26.67%)	
No	3,848 (71.25%)	4,257 (73.33%)	
Missing	613	684	
Gestational diabetes mellitus, N (%) with data			< 0.001
Yes	531 (9.02%)	381 (7.24%)	
No	5,359 (90.98%)	4,884 (92.76%)	
Missing	124	1,224	

ADHD, attention deficit hyperactivity disorder; ASD, autism spectrum disorder; ECHO, Environmental influences on Child Health Outcomes; GED, general education development; NICU, neonatal intensive care units; SD, standard deviation; sOT, synthetic Oxytocin

### Associations between sOT exposure and attention deficit hyperactivity disorder

As shown in Table 2, the adjusted association between sOT exposure and ADHD was not significant in the pooled sample (aOR 0.89; 95% CI, 0.76, 1.04). In analysis stratified by child sex, the odds ratios were not statistically significant in either male (aOR 0.89; 95% CI, 0.73, 1.07) or female offspring (aOR 0.91; 95% CI, 0.69, 1.19) (*P* = 0.83).

**Table 2:** Unadjusted and adjusted odds ratios for associations between sOT use and reported attention deficit/hyperactivity disorder (ADHD) diagnosis

	ADHD/ no ADHD	OR (95% CI)	
Total sample	1,073/11,430		
Unadjusted ^a^		0.81 (0.70, 0.94)	
Adjusted ^b^		0.90 (0.77, 1.05)	
Adjusted ^b^ + Obese		0.89 (0.76, 1.04)	
Adjusted ^b^ + GDM		0.90 (0.77, 1.05)	
Adjusted ^b^ + Obese + GDM		0.89 (0.76, 1.04)	
Males	742/5,818		
Unadjusted ^a^		0.83 (0.70, 0.99)	
Adjusted ^c^		0.90 (0.74, 1.09)	
Adjusted ^c^ + Obese		0.88 (0.73, 1.07)	
Adjusted ^c^ + GDM		0.90 (0.75, 1.09)	
Adjusted ^c^ + Obese + GDM		0.89 (0.73, 1.07)	
Females	331/5,612		
Unadjusted ^a^		0.76 (0.59, 0.98)	
Adjusted ^c^		0.92 (0.71, 1.21)	
Adjusted ^c^ + Obese		0.91 (0.70, 1.19)	
Adjusted ^c^ + GDM		0.92 (0.70, 1.20)	
Adjusted ^c^ + Obese + GDM		0.91 (0.69, 1.19)	

ADHD, attention deficit hyperactivity disorder; ASD, autism spectrum disorder; CI, confidence interval; GDM, gestational diabetes mellitus; OR, odds ratio; sOT, synthetic Oxytocin

^a^ Regression included random intercept for ECHO cohort membership

^b^ Regression adjusted for maternal age at delivery, maternal highest education level, child race, ethnicity, and sex, child birth year, gestational age at birth and large for gestational age with random intercept for ECHO cohort membership

^c^Regression adjusted for maternal age at delivery, maternal highest education level, child race, ethnicity, child birth year, gestational age at birth and large for gestational age with random intercept for ECHO cohort membership

### Associations between sOT exposure and autism spectrum disorder

The unadjusted and adjusted ORs of associations between sOT exposure during labor and delivery and ASD diagnosis are shown in Table 3. After adjusting for confounders, the aOR was 0.86 (95% CI, 0.71, 1.03) for the associations between ASD diagnosis and sOT exposure. Odds ratios were similar in male (aOR 0.81; 95% CI, 0.65, 1.01) and female offspring (aOR 0.97; 95% CI, 0.68, 1.39) (*P* = 0.42).

**Table 3:** Unadjusted and adjusted odds ratios for associations between sOT use and reported autism spectrum disorder (ASD) diagnosis

	ASD/ no ASD	OR (95% CI)
Total Sample	851/11,652	
Unadjusted ^a^		0.86 (0.72, 1.03)
Adjusted ^b^		0.87 (0.72, 1.05)
Adjusted ^b^ + Obese		0.86 (0.71, 1.04)
Adjusted ^b^ + GDM		0.87 (0.72, 1.04)
Adjusted ^b^ + Obese + GDM		0.86 (0.71, 1.03)
Males	654/5,906	
Unadjusted ^a^		0.85 (0.69, 1.04)
Adjusted ^c^		0.83 (0.67, 1.03)
Adjusted ^c^ + Obese		0.82 (0.66, 1.01)
Adjusted ^c^ + GDM		0.82 (0.66, 1.02)
Adjusted ^c^ + Obese + GDM		0.81 (0.65, 1.01)
Females	197/5,746	
Unadjusted ^a^		0.92 (0.65, 1.29)
Adjusted ^c^		0.99 (0.69, 1.42)
Adjusted ^c^ + Obese		0.98 (0.68, 1.40)
Adjusted ^c^ + GDM		0.99 (0.69, 1.41)
Adjusted ^c^ + Obese + GDM		0.97 (0.68, 1.39)

ADHD, attention deficit hyperactivity disorder; ASD, autism spectrum disorder; CI, confidence interval; GDM, gestational diabetes mellitus; OR, odds ratio; sOT, synthetic Oxytocin

^a^ Regression included random intercept for ECHO cohort membership

^b^ Regression adjusted for maternal age at delivery, maternal highest education level, child race, ethnicity, and sex, child birth year, gestational age at birth and large for gestational age with random intercept for ECHO cohort membership

^c^Regression adjusted for maternal age at delivery, maternal highest education level, child race, ethnicity, child birth year, gestational age at birth and large for gestational age with random intercept for ECHO cohort membership

### Effect modification by maternal obesity status

**Fig. 1:**
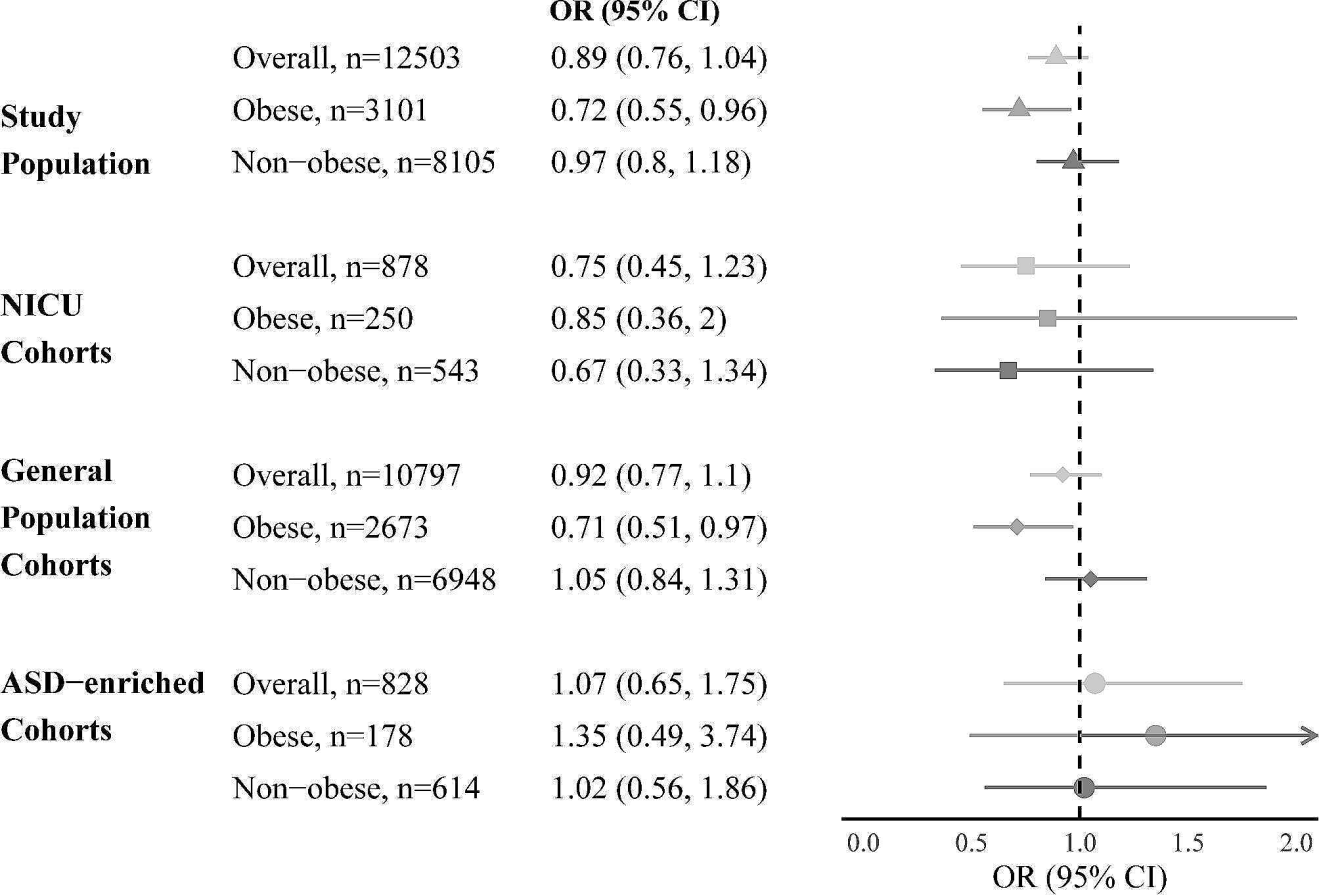
Analysis of the association of sOT and ADHD, stratified by maternal pre-pregnancy obesity. Adjusted associations between sOT exposure and attention deficit hyperactivity disorder (ADHD) stratified by obesity before pregnancy. Adjusted for maternal age at delivery, highest maternal education level, child race, ethnicity, and sex, gestational age and large for gestational age at birth, child birth year, and gestational diabetes mellitus; ASD, autism spectrum disorder; CI, confidence interval; NICU, neonatal intensive care units; OR, odds ratio; sOT, synthetic Oxytocin. ASD-enriched cohorts: *n* = 828. NICU cohorts: *n* = 878. Other cohorts: *n* = 10,797

Participant clusters grouped by maternal pre-pregnancy obesity status are shown in Table 4. In analyses adjusted for potential confounders, the interaction between sOT and maternal pre-pregnancy obesity was statistically significant for ADHD (*P* = 0.03) but was not statistically significant for ASD (*P* = 0.37). Forest plots depicting analysis of the association of sOT and ADHD, stratified by maternal obesity status, are presented in Fig. 1. Among mothers who were obese prior to pregnancy, sOT was associated with lower odds of ADHD (aOR 0.72 95% CI, 0.55, 0.96); this association was not found among children of mothers who were not obese before pregnancy (aOR 0.97; 95% CI, 0.80, 1.18).

**Table 4:** Participant clusters by pre-pregnancy obesity status

	Total	Obese	Non-obese
Overall, n	12,503	3,101	8,105
ASD cluster, n (%)	828 (6.6%)	178 (5.7%)	614 (7.6%)
NICU cluster, n (%)	878 (7.0%)	250 (8.1%)	543 (6.7%)
General population cluster (%)	10,797 (86.4%)	2,673 (86.2%)	6,948 (85.7%)

There are 1,297 (10.4%) participants missing pre-pregnancy obesity status

Overall, we did not observe significant heterogeneity in cohort-specific and cohort type-specific effect estimates for the associations between intrapartum sOT exposure and child ADHD and ASD. There was no meaningful change in effect estimates after removing each cohort and after restricting to each cohort type (NICU, ASD-enriched, general population) (Fig. 1 and Additional File 1 Figs. S2-S4).

## Discussion

In a multi-site, diverse cohort, in which 48% of mothers were administered sOT during childbirth, we found no evidence of an association between intrapartum exposure to sOT and odds of ADHD or ASD in either male or female offspring. Contrary to our hypothesis, among mothers with pre-pregnancy obesity, sOT was associated with lower odds of child ADHD diagnosis.

Our finding that intrapartum sOT exposure was not associated with adverse neurodevelopmental outcomes in the offspring is consistent with findings from several prior studies [12, 20–28]. In contrast to some of these prior studies and current results, preclinical studies suggest that sOT exposure might disrupt fetal neurodevelopment [61, 62] via cellular mechanisms such as epigenetic triggering [2, 63–65], oxytocin receptor alterations [6], DNA damage and cellular death [66, 67], complex signaling pathways [19], and transgenerational hormonal imprinting [68, 69]. Biologically plausible mechanisms that could link fetal exposure to intrapartum sOT with ADHD or ASD include excessive uterine contractility leading to decreased uteroplacental perfusion and fetal hypoxemia [18, 70–76], and especially at high cumulative doses [17] and transplacental transfer of sOT [77, 78] resulting in sOT-induced oxytocinergic signaling in the developing brain, the importance of which is suggested by the role that oxytocinergic signaling plays in the development of social behaviors that are characteristically impaired in ASD [79]. Exogenous sOT differs from the human endogenous oxytocin hormone [6, 80], and rodents exposed to sOT demonstrate altered behavioral presentations consistent with psychiatric phenotypes [81], pervasive developmental conditions [69], and enduring male specific neuroendocrine impairments, including dysfunctional cortical connectivity [71].

To our knowledge, the interaction of maternal obesity and intrapartum sOT exposure in relation to offspring neurodevelopmental outcomes has not previously been investigated. Recent reports suggest maternal weight gain and pre-pregnancy BMI may contribute to child ASD outcomes [82, 83]. Maternal obesity can lead to poor uterine contractility [84, 85], and thus impede the progression of labor and increase the likelihood of sOT exposure and exposure to higher cumulative doses of sOT [86–89]. Given these reports, we explored a potential joint effect between sOT exposure and maternal pre-pregnancy BMI on offspring neurodevelopmental outcomes in our study. Our finding that sOT was associated with lower odds of ADHD among offspring of mothers with pre-pregnancy obesity might be explained, at least in part, by confounding by indication, whereby mothers with obesity, and diminished uterine contractility, were more likely to be delivered promptly by C-section after an initial, possibly non-productive induction using sOT, thereby mitigating fetal exposure to the intense stress of labor that is typically involved during sOT exposure [90, 91]. This may also explain our observed trend of more frequent sOT childbirth intervention among mothers with pre-pregnancy obesity.

It also is plausible that in obese mothers, sOT augmentation and/or induction of labor may reduce the risk of a prolonged second stage of labor and potentially mitigate the impact of stress to the vulnerable fetal brain. Additionally, it seems possible that this exposure could mechanistically mimic the neuroprotective effect of endogenous oxytocin, as has been reported in preclinical models [92, 93].

Although our study’s findings did not confirm an association between intrapartum exposure to sOT and subsequent onset of child ADHD or ASD, the well documented routinization of sOT utilization during childbirth leaves us curious about the potential influence of this exposure on child neurodevelopmental outcomes. Synthetic oxytocin is in widespread use in the United States and globally [4, 6]. Labor induction and augmentation with sOT is one of the most prevalent clinical interventions in modern obstetric practice [86, 94]. In specific circumstances in which spontaneous labor has not begun, e.g., as pregnancies at term gestations with vertex, non-anomalous, singleton fetuses, induction of labor with sOT as compared to expectant management provides significant maternal (reduced maternal mortality, lower Cesarean delivery rate) and neonatal (reduced rate of neonatal death and meconium aspiration syndrome) benefits compared to expectant management [95–97]. Among pharmacologic agents used for labor induction and augmentation, sOT is by far the most frequently used. Furthermore, maternal obesity, and GDM are associated with higher doses of sOT during childbirth intervention [98].

For labor induction and/or augmentation, and for the management of the third stage of labor, US professional associations and the WHO recommend sOT as the uterotonic agent of choice [99–101]. This medical agent is administered intravenously, via infusion pump to provide a precise infusion rate which is adjusted based on the uterine activity (frequency and strength of contraction), fetal heart rate, and progress of labor [102]. In patients who achieve a desirable labor pattern and progress, there is no consensus about whether the sOT dose should be discontinued or continued, and consequently, sOT dosage tends to vary across birthing facilities [102]. Based on medical indication and local practices, initial sOT dosage varies from 0.5 to 6 milliunits/minute and the maximum dose varies between 16 and 64 milliunits/minute. Per this protocol, sOT is administered continuously until which point uterine contractions are deemed inefficient to reliably expel the fetus, and labor is declared a “failure to progress,” warranting a Cesarean Sects. [62, 103].

### Strengths and limitations

A chief limitation of our study was our lack of information on indications for childbirth intervention with sOT (specifically, the clinical indication for labor induction or augmentation), length of labor, mode of delivery (e.g. vaginal or C-section), and sOT dosage administered to laboring mothers during offspring delivery. We defined sOT exposure as a binary category, so we were unable to assess a potential dose-response association, or threshold effects. Findings from a study by Soltys et al. (17) are consistent with the concept that the strength and direction of the relationship of sOT and ASD varies across a range of sOT doses; specifically, low dose/short duration sOT exposure was associated with a statistically non-significant decrease in the odds of ASD, moderate dose/duration was associated with a non-significant increase in odds of ASD, and high dose/long duration exposure was associated with an increase in odds of ASD among male offspring. Our use of binary exposure limited the opportunity to assess such dose-dependent associations, leaving us questioning a potential dose-response influence on our results.

Given the limitations of the current study, and the fact that the main non-null finding was unexpected, replication of our analyses in other cohorts with clinical data related to indication for and dosage of intrapartum sOT is needed before drawing conclusions about associations between intrapartum sOT exposure and neurodevelopmental outcomes in the offspring.

Another potential limitation of our study is that child diagnoses of ADHD or ASD were based on parent report of physician diagnosis, rather than a rigorous assessment by clinicians with expertise in diagnosing these specific neurodevelopmental conditions, which could have led to misclassification regarding our outcomes.

Despite these limitations, our study had some notable strengths including a large, diverse, multi-site study cohort, which allowed us to derive precise estimates of associations, adjust for confounders, and explore effect measure modification by maternal pre-pregnancy obesity. Secondly, this was the first known endeavor which assessed the interaction between intrapartum sOT exposure and maternal BMI on child neurodevelopmental outcomes.

## Conclusions

In a sample from the ECHO cohort, we found no evidence of an association between intrapartum sOT exposure and ADHD and ASD in the offspring. Instead, we observed an unexpected association between intrapartum sOT exposure and decreased odds of child ADHD among women with pre-pregnancy obesity. We observed use of intrapartum sOT in nearly half our sample, and more frequently among mothers with pre-pregnancy obesity. The unknown complexities, and under-investigated mechanisms and pathways of intrapartum sOT as weighed against the sensitivity of the still developing fetal brain provides a robust opportunity for future exploration regarding this early exposure.

### Electronic supplementary material

Below is the link to the electronic supplementary material.

Supplementary Material 1

Supplementary Material 2

## Data Availability

Select de-identified data from the ECHO Program are available through NICHD’s Data and Specimen Hub (DASH). Information on study data not available on DASH, such as some Indigenous datasets, can be found on the ECHO study DASH webpage.

## Abbreviations

ADHD: Attention deficit hyperactivity disorder
aOR: Adjusted odds ratio
ASD: Autism spectrum disorder
BMI: Body mass index
CI: Confidence Interval
ECHO: Environmental influences on Child Health Outcomes
GDM: Gestational diabetes mellitus
INTERGROWTH-21: International Fetal and Newborn Growth Consortium for the 21st Century
ICD: International Classification of Disease
LGA: Large for gestational age
NICU: Neonatal intensive care units
sOT: Synthetic oxytocin
