# Supplementary material for: Intrapartum exposure to synthetic oxytocin, maternal BMI, and neurodevelopmental outcomes in children within the ECHO consortium
Source: J Neurodev Disord. 2024 May 26;16:26. doi: 10.1186/s11689-024-09540-1 (PMC11128127; doi:10.1186/s11689-024-09540-1)
Supplement: Supplementary file 1 — Supplementary Material 1 [file 11689_2024_9540_MOESM1_ESM.docx]

**Intrapartum Exposure to Synthetic Oxytocin, Maternal BMI, and Neurodevelopmental Outcomes in Children within the ECHO Consortium**

**ADDITIONAL FILE 1**

**Table S1** Supplemental Table 1 Distribution of ASD, ADHD, and sOT administration in ECHO cohort groups included in analyses

**Table S2** Number of participants in individual cohorts

**Figure S1** Inclusion in the ECHO analysis.

**Figure S2** Interaction for obesity and ASD (NS).

**Figure S3** Leave one out analyses for ASD.

**Figure S4** Leave one out analyses for ADHD.

***Abbreviations*:** ADHD, attention deficit hyperactivity disorder; ASD, autism spectrum disorder; ECHO, Environmental influences on Child Health Outcomes; NS, not significant; sOT, synthetic Oxytocin.

| **Table S1** Distribution of ASD, ADHD, and sOT administration in ECHO cohort groups included in analyses | | | | |
| --- | --- | --- | --- | --- |
|  | **ASD / no ASD** | **ADHD / no ADHD** | **sOT / no sOT** | **Total** |
| ASD cluster (N=3) | 389/379 | 84/535 | 557/211 | 768 |
| NICU cohorts (N=6) | 67/406 | 85/376 | 87/389 | 476 |
| General population (N=29) | 129/5020 | 409/4759 | 2389/2806 | 5195 |

ADHD, attention deficit hyperactivity disorder; ASD, autism spectrum disorder; ECHO, Environmental influences on Child Health Outcomes; NICU, neonatal intensive care units; sOT, synthetic Oxytocin.

**Table S2** Number of participants in individual cohorts

| **Cohort** | **ASD Analysis**  **(N=6390)** | **ADHD Analysis**  **(N=6248)** |
| --- | --- | --- |
| 1 | 39 | 39 |
| 2 | 5 | 5 |
| 3 | 15 | 15 |
| 4 | 12 | 13 |
| 5 | 72 | 71 |
| 6 | 191 | 189 |
| 7 | 70 | 93 |
| 8 | <5 | <5 |
| 9 | 52 | 52 |
| 10 | 141 | 141 |
| 11 | 899 | 896 |
| 12 | 734 | 734 |
| 13 | 480 | 480 |
| 14 | 62 | 63 |
| 15 | 762 | 613 |
| 16 | 24 | 24 |
| 17 | 1166 | 1166 |
| 18 | 21 | 22 |
| 19 | 145 | 132 |
| 20 | 103 | 103 |
| 21 | 56 | 56 |
| 22 | 451 | 451 |
| 23 | <5 | <5 |
| 24 | 5 | 5 |
| 25 | 19 | 19 |
| 26 | 257 | 257 |
| 27 | 229 | 227 |
| 28 | 33 | 31 |
| 29 | 10 | 10 |
| 30 | 52 | 52 |
| 31 | 6 | 6 |
| 32 | 27 | 28 |
| 33 | 27 | 29 |
| 34 | 30 | 31 |
| 35 | 6 | 6 |
| 36 | 36 | 36 |
| 37 | 138 | 138 |
| 38 | 10 | 10 |

ADHD, attention deficit hyperactivity disorder; ASD, autism spectrum disorder


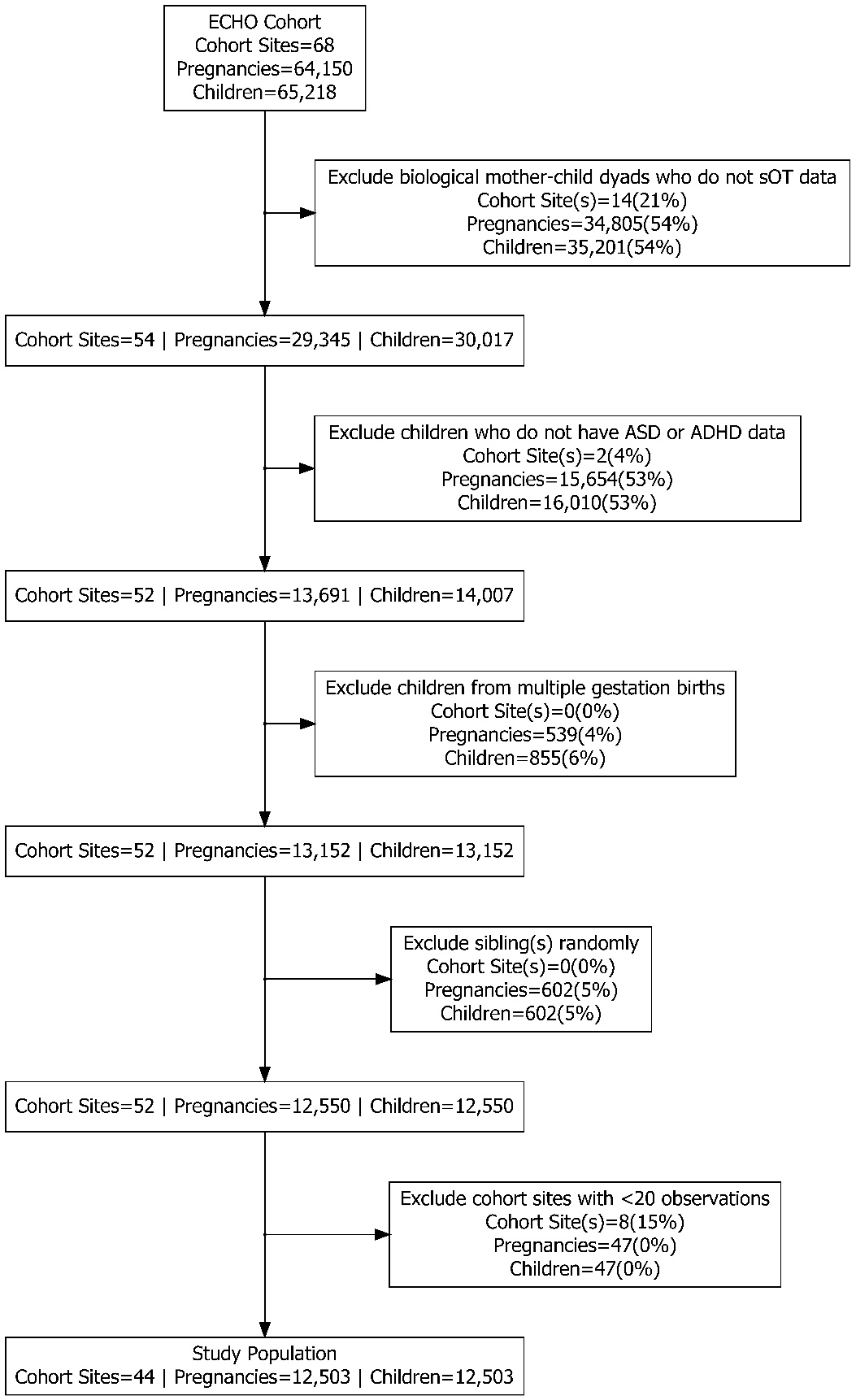


**Fig. S1** Inclusion in the ECHO analysis.


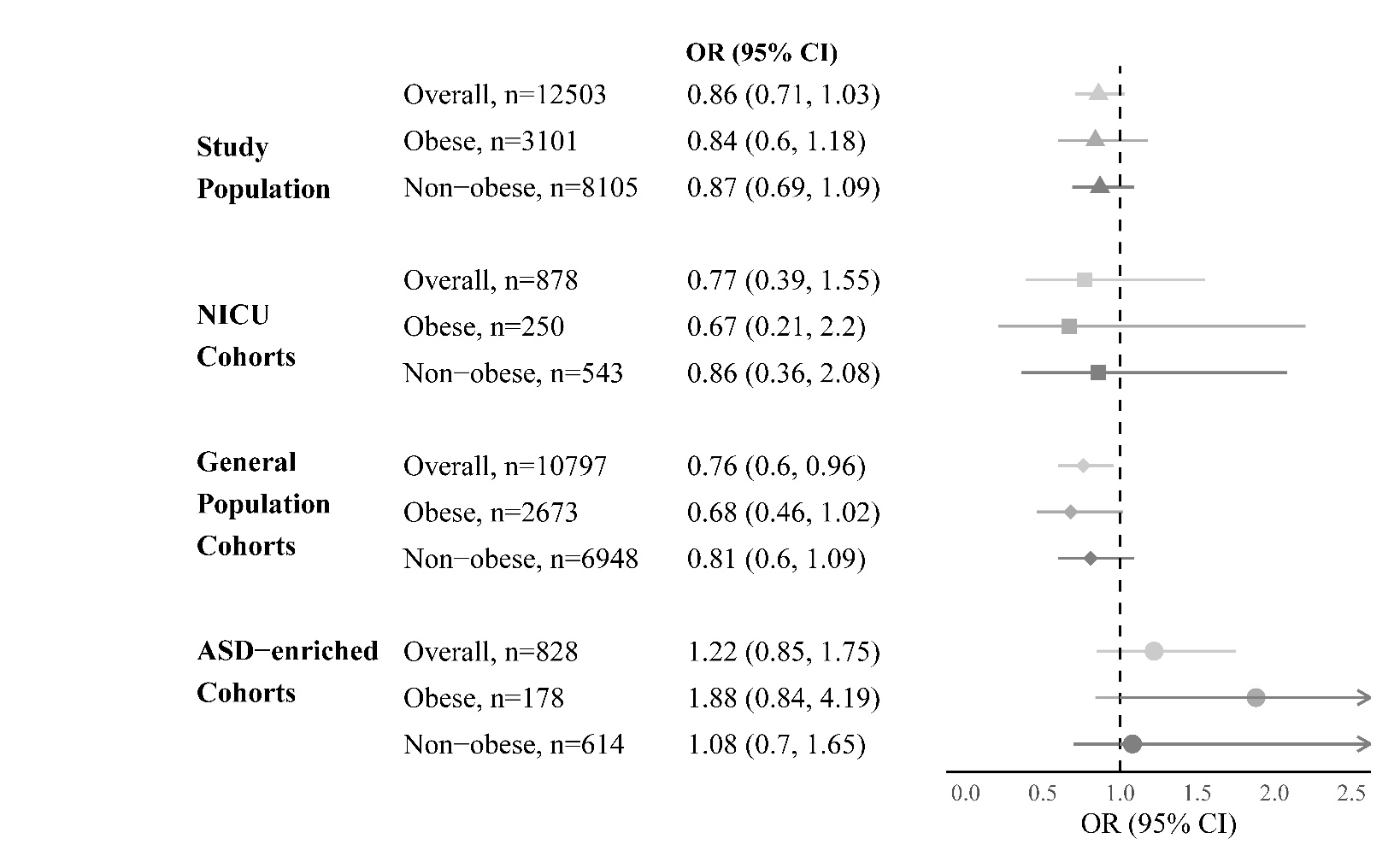


**Fig. S2** Interaction for obesity and ASD (NS).


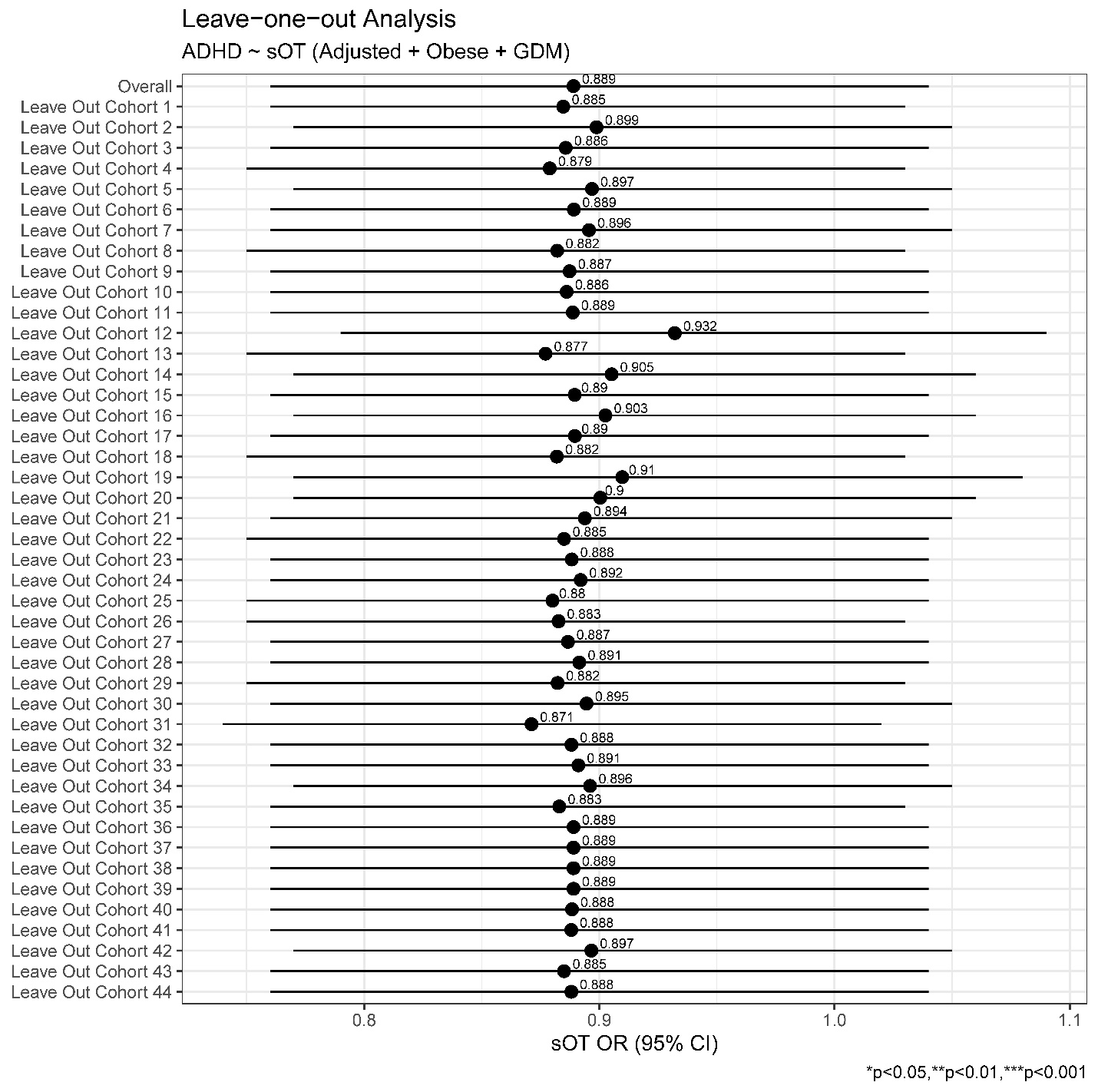


**Fig. S3** Leave one out analyses for ASD.


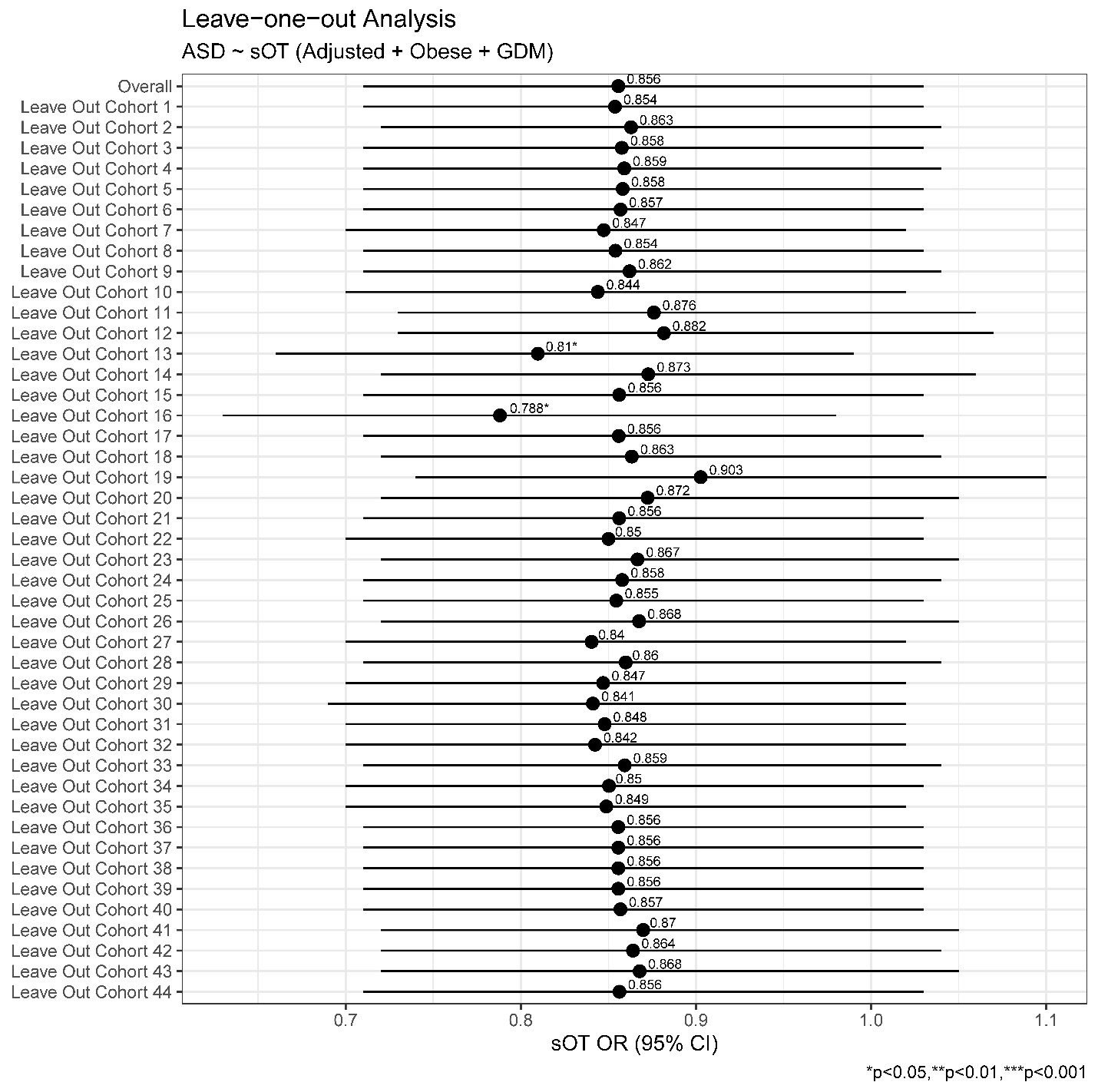


**Fig. S4** Leave one out analyses for ADHD.
