# Supplementary material for: Intrapartum exposure to synthetic oxytocin, maternal BMI, and neurodevelopmental outcomes in children within the ECHO consortium
Source: J Neurodev Disord. 2024 May 26;16:26. doi: 10.1186/s11689-024-09540-1 (PMC11128127; doi:10.1186/s11689-024-09540-1)
Supplement: Supplementary file 2 — Supplementary Material 2 [file 11689_2024_9540_MOESM2_ESM.docx]

Lisa Kurth, PhD

University of Colorado School of Medicine

Anschutz Medical Campus

13123 E. 16th Ave. B065

Aurora, CO 80045

Phone: 720-777-2715

Email: [lisa.kurth@cuanschutz.edu](mailto:lisa.kurth@cuanschutz.edu)

January 25, 2023

Joseph Piven, MD

Editor-in-Chief

*Journal of Neurodevelopmental Disorders*

University of North Carolina

Chapel Hill, NC

Dear Dr. Piven:

On behalf of my co-authors, I am pleased to submit our original research manuscript titled “Intrapartum Exposure to Synthetic Oxytocin, Maternal BMI and Neurodevelopmental Outcomes in Children within the ECHO Consortium” to be considered for publication in *Journal of Neurodevelopmental Disorders.*

Synthetic oxytocin (sOT) is widely used as a first line approach to induce and/or augment labor in pregnant women. Despite the increasing frequency of sOT use, very few large studies have characterized the relationship of intrapartum sOT and child neurodevelopmental outcomes. Some existing studies report possible associations between sOT and risk of attention deficit hyperactivity disorder (ADHD) and autism spectrum disorder (ASD). To our knowledge, no prior large epidemiologic studies involving US births have investigated the association of intrapartum sOT, and the modifying effects of maternal obesity, with child neurodevelopmental outcomes.

This study used data from the Environmental influences on Child Health Outcomes (ECHO) program (N=6439) to evaluate the association between perinatal sOT and offspring ADHD and ASD. In addition, maternal obesity and child sex were evaluated for effect modification. In this large diverse sample, we found no convincing evidence of an association between perinatal exposure to sOT and odds of ASD or ADHD. We did, however, realize an unexpected, significant association between sOT exposure and a reduced risk of ADHD specific to offspring of obese mothers.

This manuscript has not been previously published, nor is it under consideration elsewhere. All authors meet all the criteria for authorship and have reviewed and approved the manuscript for submission. The authors have no conflicts of interest relevant to this article to disclose.

Thank you for your consideration.

Sincerely,

Lisa Kurth, PhD
